# The Attentional Bias in Current and Former Smokers

**DOI:** 10.3389/fnbeh.2019.00154

**Published:** 2019-07-10

**Authors:** Marianna Masiero, Claudio Lucchiari, Patrick Maisonneuve, Gabriella Pravettoni, Giulia Veronesi, Ketti Mazzocco

**Affiliations:** ^1^Department of Biomedical and Clinical Sciences (DIBIC), Luigi Sacco, University of Milan, Milan, Italy; ^2^Applied Research Division for Cognitive and Psychological Science, European Institute of Oncology (IEO), IRCSS, Milan, Italy; ^3^Department of Philosophy, University of Milan, Milan, Italy; ^4^Division of Epidemiology and Biostatistics, European Institute of Oncology (IEO), IRCSS, Milan, Italy; ^5^Department of Oncology and Emato-Oncology (DIPO), University of Milan, Milan, Italy; ^6^Division of Thoracic and General Surgery, Humanitas Research Hospital, Rozzano, Italy

**Keywords:** cigarette smoking, attentional bias, former smokers, implicit cognition, impulsiveness, inhibition

## Abstract

Attentional bias has been defined as the propensity of a person to allocate selective attention automatically to salient cues (Field and Powell, 2007). In the case of smoking, this bias implies that smokers are implicitly attracted by smoking-related stimuli, which produce behavioral, memory, and emotional effects (Volkow et al., 2006; Giardini et al., 2009). In more detail, scientific evidence pointed out that smoking is strongly supported by attentional bias that activates craving and urgency to smoke a cigarette. However, poor and conflicting data are available regarding the role of this cognitive bias on former smokers. The main aim of this study is to explore the occurrence of the attentional bias on of both current and former smokers, also with the aim to identify associations with behavioral, psychological and cognitive characteristic of participants. We collected data on 245 current, volunteers (male 50.6%; female 49.4%) aged 54.81 (SD = 14.352, range = 18–63), divided in current smokers (98), former smokers (102) and non-smokers (45). A combination of neuropsychology tests (Emotional Smoke Stroop Task and Go/no-Go task), and standardized questionnaires [Behavioral Inhibition System-Behavioral Approach System (BIS-BAS), Fagerström Test for Nicotine Dependence (FTND), Barratt Impulsiveness Scale, Motivational questionnaire] were used to assess the attentional bias, psychological variables, and smoking-related characteristics. Responses at the Emotional Smoke Stroop task revealed that current and former smokers are actually slower than non-smokers are when facing smoking cues, while performances at other Stroop conditions and at the Go/no-Go task are not statistically different. These results confirmed the occurrence of the attentional bias in current smokers, and above all points out that the same effect is present in former smokers. We found only small and selective correlations between attentional bias and psychological variables (e.g., impulsiveness and inhibition). In particular, impulsivity is not directly associated with the AB intensity. Also, smoking characteristics (e.g., years of smoking and dependence level) and the length of the period of abstinence do not seem to modulate implicit cognition of smoking cue. Our data support the idea that the attentional bias may be considered relevant in sustaining smoking and favoring relapse.

## Introduction

Growing evidence of the negative effects of tobacco cigarette smoking on health has had little impact on the real extent of this phenomenon and in promoting solutions (Morgan et al., 2011). The main issue is concerned with the relapse. For example, Hughes et al. (2004) reported that 85% of former smokers are more likely to relapse after 1 year from quitting. This is also true for those who followed a cessation program (Yong et al., 2018) and even after a continued period of abstinence (Kerr et al., 2011). Probably, the limited effectiveness of smoking cessation programs depends on individual biological, psychological and cognitive factors, which effect smoking initiation and maintenance (Kale et al., 2018).

From a general point of view, addictions are modulated by different mechanisms, which include: drug-related Pavlovian and instrumental reinforcement (Everitt and Robbins, 2005); biases toward drugs-cues (Grant et al., 1996; Hester et al., 2006); the effect of rewards and reward expectancy on decision-making (Grant et al., 2000; Bechara et al., 2002; Stout et al., 2005; Goldstein et al., 2007; Wrase et al., 2007); cognitive monitoring and inhibition processes (Kaufman et al., 2003; Forman et al., 2004). However, a pivotal role in the adoption on unhealthy behaviors (such as smoking, alcohol consumption and an unhealthy diet) is the biased cognitive processing of salient cues (Kakoschke et al., 2017).

In particular, the attentional bias (AB; Williams et al., 1988) seem to be particularly relevant, as shown by several studies in different areas such as anxiety disorders (Pool et al., 2016), food consumption (Deluchi et al., 2017), alcohol abusers (Manchery et al., 2017) and cocaine users (Marks et al., 2016). A plethora of studies reported this effect to be present also within smokers (Bradley et al., 2003; Drobes et al., 2006; Munafò et al., 2003; Waters et al., 2003b).

With regards to this, the study of smoking-related cognitive biases offers additional opportunities to develop tailored and effective anti-smoking programs (Mühlig et al., 2016) based on the assessment of cognitive and psychological processes engaged in smoking behavior (Kondylakis et al., 2012; Fioretti et al., 2016; Gorini et al., 2016; Lucchiari et al., 2016; Masiero et al., 2019).

Currently, two main theories are used to explain the activation of cognitive biases in smoking behavior: the incentive salience theory (IST; Robinson and Berridge, 1993) and Pavlovian conditioning (PC). According to the IST, the individual reactivity to drug-related cues (e.g., objects or situations directly or indirectly associated with cigarettes, e.g., an ashtray, a lighter, coffee after lunch, a group of friends) plays a pivotal role in smoking behavior. Smokers tend to allocate selective attention automatically to smoking cues (Field and Powell, 2007). These cues can elicit a physiological response similar to the one activated by nicotine, thus producing memory, emotional and perceptive effects that facilitate the maintenance of smoking (Volkow et al., 2006; Giardini et al., 2009).

According to the PC theory, instead, when a person develops an addiction, the related objects or events (e.g., a cup of coffee) undergo a change in their cognitive status: from the original neutral status, they become conditioned motivational triggers (Marlatt, 1990; Di Chiara, 2000; Caretti and La Barbera, 2010). This process stimulates the compulsive need to consume substances (e.g., nicotine), just because of the activating effect of the conditioned event (Giardini et al., 2009). Consequently, drug-related cues are able to activate automatic and implicit processes that stimulate craving and the urgency to smoke.

The AB intensity depends on a variety of factors, some linked to personal characteristics and other to contextual factors. For example, Di Chiara (2000) affirmed that cigarette smokers are particularly sensitive to incentives in the early phase of smoking behavior, while the same incentives have a lower or no impact in chronic smokers (Mogg et al., 2005). Furthermore, Drobes et al. (2006) reported that susceptibility to the AB is strictly linked to nicotine dependence (Drobes et al., 2006). In particular, smokers who have a high level of nicotine dependence are more susceptible to AB than smokers who have low nicotine dependence. In addition, reactivity to smoking-related cues (SC) is modulated by gender, as women seem to be more affected. On this point, Field and Cox (2008) affirmed that women are more vulnerable to conditioned factors of smoking behavior (Field and Duka, 2004; Saladin et al., 2012) suggesting that these factors are a possible roadblock for women to stop smoking (Smith et al., 2016). Other authors observed that women who smoke have a specific “neurocognitive profile” characterized by impairments in sustained attention and control of impulsivity that may facilitate both smoking initiation and stabilization of this behavior (Yakir et al., 2007). The AB in cigarette smokers, similarly to alcohol abusers, seems to be amplified by the emotional distress, excessive alcohol consumption, withdrawal, drug-related stimuli, and the perception that the opportunity to consume the substance is imminent (Field et al., 2014). Finally, some evidence suggests that impulsivity affects the strength of AB, since the more people are impulsive the greater is their bias (Field and Cox, 2008). Actually, cognitive control functions and impulsivity may be of particular relevance, since selective impairments may predispose some individuals to impulsive use of the drug. For example, a high level of impulsivity in 10–12-year-olds seems to predict drug use at the age of 19 (Tarter et al., 2003), suggesting an important role on this dimension in the transition from recreational to dependent use. Similarly, Perkins et al. (2008) reported that personality traits related to impulsiveness, for example, novelty seeking and response disinhibition, are associated with sensitivity to nicotine, including reinforcement and reward (Perkins et al., 2008). In addition, the evidence of higher impulsiveness supports the transition from occasional to chronic smoking (Hu et al., 2006; DiGirolamo et al., 2016).

The AB has an important effect on action. It seems to facilitate the repetition of smoking (Tiffany, 1990) and the development of habits. The same mechanism is also effective when a smoker attempts to quit. For example, former smokers may feel the desire to smoke when they are found in a place where they used to smoke in the past (e.g., waiting for a bus, watching TV, at the coffee vending machine and so on), potentially modulating individual motivation by the mediation of the brain wanting system, which includes the dopaminergic mesolimbic circuit (Berridge and Robinson, 2016). In particular, neuro-imaging studies highlighted that SC are able to activate smokers’ neural mechanisms linked to gratification (Garavan et al., 2000; Wexler et al., 2001). These studies confirmed the role of dopamine in the shell of the nucleus accumbens: an event (e.g., smoking after a cup of coffee) is marked by a discharge of dopamine in the nucleus accumbens, which integrates affective and contextual attributes of a learning experience due to the input from the amygdala and hippocampus (Kerfoot and Williams, 2018).

A few studies investigated the presence of AB in former smokers, some reporting a similar attention reactivity to smoking cues in current and former smokers, and some other disconfirming this view. In particular, Ehrman et al. (2002) found that former smokers suffer from an AB in a 500 ms visual probe task, but the intensity of this bias was considered intermediate with respect to current smokers. However, in this study, the former smoker sample was small and with a very short abstinence time (only 1 week). Munafò et al. (2003) compared 43 current smokers, 22 former smokers and 30 never-smokers using an Emotional Smoke Stroop task. They found smoking-related interference (AB) only in current smokers since former smokers and non-smokers reported similar reaction time to smoking-related words. In addition, authors reported that AB in current smokers was associated with the personality trait of sensibility to reward. Peuker and Bizarro (2014), in a sample of 60 former smokers divided into three different abstinence time (recent, intermediate and prolonged abstinence) found longer reaction times at a visual probe task in recent and intermediate former smokers (Peuker and Bizarro, 2014). Another recent study by Rehme et al. (2018) tested 38 former smokers and 34 current smokers using an Emotional Smoke Stroop task (Rehme et al., 2018). They found that AB affected both former and current smokers in visual orienting to smoking pictures and that this effect was negatively related to nicotine dependence in current smokers. Munafò et al. (2008) suggested that the presence and the persistence of the AB might be linked to a subgroup of formers smokers with a particular genetic configuration. Finally, Nestor et al. (2011) explored the neural correlates of attentional bias in non-smokers, current smokers and former smokers while their neural activity was being recorded by means of functional magnetic resonance imaging (fMRI). The task required subjects to respond to the color of a border framing neutral, emotionally salient or smoke-related pictures. Authors found only a small behavioral effect linked to AB in former smokers, but importantly they found a remarkable difference in cortical activations. On the one hand, compared to former smokers, current smokers showed a decreased activity in higher-order cortical areas in favor of subcortical regions (Nestor et al., 2011). On the other hand, former smokers showed an opposite pattern, with increased prefrontal cortical activity. The authors interpreted such findings as a tendency of former smokers to adopt more top-down brain strategies when facing smoke-related stimuli (Tuma and Pratt, 1982). Taken all together, data about the presence of AB in former smokers are still poorly supported and not convergent (Field et al., 2014; Rehme et al., 2018).

According to this background, the primary aim of the current study is to investigate the presence of the AB in former smokers and to compare this effect on a sample of current smokers. In particular, we were interested in former and current smokers with a long history of cigarette smoking and, in the case of former smokers, with consolidated abstinence (being abstinence for at least 1 year). Although our main aim is to compare smokers and former smokers, we also collected data on a sample of people who have never smoked, so to have a benchmark for the variables we used.

We defined AB as a significant increase in response time to SC during the Emotional Smoke Stroop task with respect to the response time to color coherent (baseline) condition. Therefore, we hypothesized that the effect of the AB, i.e., the smoking-related latency, is higher in smokers than former smokers, while non-smokers should not be affected by SC, showing latencies similar to the coherent condition.

We also expected to find differences with regard to implicit processes. In fact, the AB may be considered a measure of cognitive implicit processes (Kakoschke et al., 2017), thus involving not only selective attention but also inhibitory control. Inhibitory control is a core component of executive functioning and is defined as the ability to inhibit a motor response that has already been initiated, the ability to suppress interfering stimuli, impulsiveness as well as approach and avoidant attitudes (Everitt and Robbins, 2005). Consequently, it is possible to assume that smoking addiction is generally sustained by implicit appetitive processes potentially triggered by smoking cues even when they are outside awareness. We then hypothesized that people with low inhibitory control (as measured by Go/no-go task) might be particularly affected by a bias toward smoking cues (de Wit, 2009). Finally, we wanted to test if impulsivity and approach attitudes could modulate the AB intensity. Although there are contradictory data (Coskunpinar and Cyders, 2013), we hypothesized that more impulsive individuals are particularly affected by AB.

## Materials and Methods

### Participants

Two-hundred and forty-five participants (male 50.6%; female 49.4%) aged 51.79 (SD = 6.258, range = 34–63) were recruited. The sample of the present study consisted of 98 current smokers (40%), 102 former smokers (41.7%) and 45 non-smokers (18.3%).

Smokers and former smokers were recruited within the participants at the Continuous Observation of SMOking Subjects I (COSMOS I), a screening program for early detection of lung cancer using a low-dose computed tomography (CT) scan, run at the European Institute of Oncology (IEO) in Milan, Italy. Detailed information about COSMOS I protocol has been published elsewhere (Veronesi et al., 2008). Participants were contacted by a researcher, who described the study and made the first interview. The informed consent form was then provided with full details. In case of acceptance, data recording was run at the hospital, after the CT in order to reduce possible confounding variables due to anxiety, fear or rush.

Non-smokers were recruited through advertising on the Hospital and University sites and with the collaboration of the IRIDe—Interdisciplinary Research Centre on Decision-making research of the University of Milan. Non-smoker participants were first contacted by phone and invited for the first interview at an IRIDe office. Then, the study was presented, and full details about the experimental setting provided. Data collection was completed in a second meeting.

Inclusion criteria for smokers and former smokers were: being enrolled at the COSMOS I program; not having a history of neurological and psychiatric diseases; having smoked for more than 10 years at least 10 cigarettes a day and/or being abstinence at least from 1 year; agreed to sign the informed consent. With regard to non-smokers: no history of neurological or psychiatric conditions; no history of antismoking interventions. Participants were volunteers and they could withdraw their consent at any time during the study.

The Ethical Committee of the European Institute of Oncology (IEO) approved the study. All enrolled participants read, filled in and signed the informed consent form. The study was in accordance with the principles stated in the Declaration of Helsinki (59th WMA General Assembly, Seoul, 2008).

### Procedure

Data collection consisted in two consecutive phases. In the first one, in order to obtain a psycho-cognitive profile, each participant received a set of article and pencil questionnaires to be carefully read and filled out in a quiet room. Overall, the mean time required to complete questionnaires was about 20 min per participant. Then, in the second phase, a computerized short neuropsychological battery was delivered by the use of the Millisecond Inquisit Lab software (version 4.0). Participants had the time to familiarize with the software for 5 min. A further familiarization period was also provided before every single test within the battery. At the end of the assessment, each participant took part in a debriefing section, during which smoking cessation strategies were also discussed (about 15 min).

### Instruments

#### Questionnaires

##### Behavioral Inhibition System/Behavioral Approach System (BIS/BAS) Questionnaire

A 20-item self-administered questionnaire assessing the affective reaction to punishment and reward. Answers were assessed using a five-point Likert scale (from 1 = “it does not describe me at all” to 5 = “it describes me completely”). The questionnaire includes six subscales: Behavioral inhibition system (BIS); Behavioral activation system (BAS); Drive; Fun seeking; Reward responsiveness (Carver and White, 1994). BIS/BAS is a valid and reliable measure with Cronbach alpha ranging from 0.67 to 0.84 in several studies (e.g., Leone et al., 2001; Cerutti et al., 2012).

##### Fagerström Test for Nicotine Dependence (FTND)

A 6-item self-administered questionnaire assessing nicotine dependence. The score range is from 0 to 10 points. It includes four categories: low dependence (0–2); middle (3–4); strong (5–6); very strong (7–10; Heatherton et al., 1991). The Italian version was previously validated (Fekketich et al., 2008).

##### Barratt Impulsiveness Scale (BIS-11)

A 30-item self-administered questionnaire that assesses impulsiveness trait on 4-point scales (never, occasionally, often, and always). It is made by three subscales: attentional impulsiveness, motor impulsiveness, and non-planning impulsiveness. Higher values indicate higher impulsivity (Patton et al., 1995). The Cronbach’s alpha of the Italian version was found to be 0.79, while a 2-month test-retest reliability coefficient was 0.89 (Fossati et al., 2001).

##### Motivational Questionnaire

A 4-item self-administered questionnaire aimed at assessing motivation to quit. The total score classifies the patient into 1 out of 4 motivational categories (from “not ready to quit” to “highly motivated”). Higher values are suggestive of higher motivation (Marino, 2002).

### Neuropsychological Measurements

#### Go/no-Go Task

This task measures inhibition and response control (Costantini and Hoving, 1973). Participants face stimuli on which they have to take a binary decision (go or no-go). In the version we used, participants were asked to press the spacebar when they saw a green rectangle (go), but to refrain from pressing the spacebar when they saw a blue rectangle (no-go). The blue and green rectangles could be vertical or horizontal with different probabilities of being green or blue. The vertical rectangle had a high probability (805) of being green (a go trial) and the horizontal rectangle had a high probability (80%) of being blue (a no-go trial). Participants get information about the orientation of the rectangle (cue) shortly before the color of the rectangle is revealed. In this way, subjects must overcome the acquired go response in order to inhibit the response if a no-go target is subsequently displayed (Fillmore et al., 2006). An equal number of vertical and horizontal cues were presented before an equal number of go and no-go target stimuli.

The task measure failure of response inhibition (the proportion of no-go targets in which a subject failed to inhibit a response) and speed of response execution (reaction times to go targets).

#### Emotional Smoke Stroop Task

This task was adapted from the original version of the Stroop Task (Stroop, 1992) in order to measure AB in smokers. Each participant was asked to name the ink color of the word that appears on the PC monitor, neglecting the semantic content of the word. The task includes four categories of cues: SC (e.g., tobacco, nicotine, tar, package, cigarette, smoking, filter, asthma, cancer, tobacconist, breath, pollution, bronchitis, coffee, lung, bad habit, dependence, cigar), neutral cues (NC; e.g., butterfly, computer, wood, dream, book, vase, bed, flower, iron, pizza, dish, food, spider, water, hot, table, box, door, rug, alcohol) congruent color and incongruent color words (Waters et al., 2003a,b). The emotional cues will be mentioned as “smoking-related” cues.

### Statistical Analysis

Descriptive statistics were used to describe the sample’s characteristics. The AB was calculated using the latency time between the presentation of the stimulus and the verbalization of the ink color for SC, NC, congruent color words (CC), and incongruent color words (IC). Inhibition was instead assessed using reaction times and the number of errors in the Go/No Go task.

More specifically, in order to avoid biases due to the different sample sizes, we ran a one-way analysis of variance (ANOVA) using smoking status (current smokers, former smokers, and non-smokers) as a fixed factor and latencies at smoking-related words as a dependent variable. Since we defined AB as the difference between the response latency to a SC and the latency at the congruent stimuli (CC), we computed a new variable (AB) subtracting the mean latency at CC from SC. Therefore, a second one-way ANOVA test was run on this variable. When the interaction between variables was considered of interest, mixed design ANOVAs were used to compare the latencies at different tasks between groups (current smokers, former smokers). Bonferroni corrections for multiple paired comparisons were applied.

The Pearson coefficient was used in order to assess the association between impulsiveness, BAS and BIS, nicotine dependence, the number of cigarettes per day, and the age of the first cigarette. Bonferroni correction for the *p* values was used also for correlations. Finally, two linear regressions were performed for current smokers and former smokers. In the first linear regression for current smokers, AB index was used as the criterion variable, while impulsiveness, years of smoking, number of daily cigarettes, dependence level, BIS/BAS subscales and age as predictors. In the second linear regression for former smokers, AB index was included as criterion variable and impulsiveness, number of years as smokers, number of years from the interruption, BIS/BAS subscales, and age as predictors. All the analyses were performed using the SPSS package (version 23.0, IBM, USA, 2014).

## Results

Smoking characteristics of current and former smokers are reported in Table 1, while Table 2 shows BIS/BAS and BIS-11 scores. Considering both current and former smokers, the mean number of cigarettes smoked per day was 21.82 (SD = 12.03, range = 1–80), the mean number of years of regular smoking was 36.56 (SD = 14.52) and the mean age of the first cigarette was 21.72 (SD = 8.29, range = 6–59).

**Table 1:** Mean standard deviation values and analysis of variance (ANOVA) *p*-values of participants’ characteristics.

	Current smokers		Former smokers		
Descriptive statistics	*M*	SD	*M*	SD	
Daily cigarettes*	18.745	10.521	24.434	12.643	0.011
The age of the first cigarette	20.79	8.561	22.46	8.054	0.126
Number of years of regular smoking	41.653	9.173	33.781	16.166	0.211
Number of the years from the interruption	−	−	7.42	5.525	
Nicotine dependence level	5.23	3.213			
Motivational level	10.74	3.334			

*The values of mean and standard deviation for former smokers refer to their past smoking history.

**Table 2:** Mean values, standard deviations and ANOVA *p*-values of participants’ impulsiveness and activation/inhibition.

	smokers		Former smokers		Non-smokers		
Descriptive statistics	*M*	SD	*M*	SD	*M*	SD	*p*
***BIS-11***							
Attentional impulsiveness	15.69	3.24	15.77	3.776	15.66	3.153	0.890
Motor impulsiveness	19.71	3.974	19.83	5.018	19.16	2.838	0.750
Non-planning impulsiveness	25.36	4.863	24.5	5.421	25.02	5.438	0.155
Total score	60.76	9.258	60.1	11.974	59.84	8.927	0.455
***BIS-BAS***							
Bis	21.35	5.134	22.76	4.454	23.42	5.544	0.380
Bas	39.37	9.147	42.31	8.176	42.58	7.554	0.115
Reward responsiveness	18.68*	4.313	19.75	3.372	20.46	3.53	0.137
Drive	10.89	3.618	12.04	3.368	11.84	2.874	0.112
Fun seeking	9.89	3.733	10.52	3.545	10.28	3.654	0.421

Note: Go/no-Go task (latencies and errors), *p < 0.05.

An ANOVA test was run to compare BIS/BAS and BIS-11 among groups. Current smokers resulted lower in reward responsiveness (*F*_(2,244)_ = 3.101, *p* = 0.0041), with no further differences in BIS, BAS, and BIS-11 dimensions.

We considered response inhibition as the main dependent variable here, so we focused on reaction times and failure of response inhibition (false alarm) calculated as the number of errors on No-Go trials divided by the total number of No-Go trials.

No differences between the three groups were found. In particular, the false alarm rate was 0.443 for current smokers, 0.395 for former smokers and 0.492 for non-smokers (*F*_(2,244)_ = 0.722, *p* = 0.495). The mean latency was 677.12 ms for current smokers, 683.27 ms for former smokers and 685.84 ms for non-smokers (*F*_(2,244)_ = 0.525, *p* = 0.594).

### Emotional Smoke Stroop Task Latency

**Figure 1:**
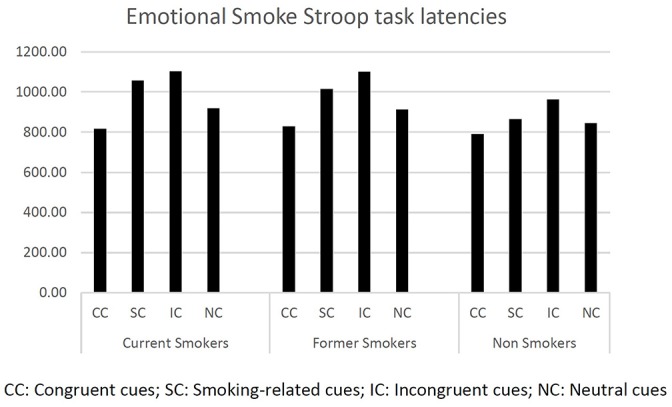
Latency mean values of each group at the Emotional Smoke Stroop Task.

First, we computed descriptive statistics on response latencies (see Figure 1).

We first ran one-way ANOVAs on latencies (dependent variable) at each Stroop condition (CC, IC, SC, NC) to test differences between current smokers, former smokers, and non-smokers (fixed factor). We found a significant difference between groups only for the smoking cues (*F*_(2,244)_ = 3.822, *p* < 0.029). Bonferroni pairwise comparisons showed current smokers and former smokers to be significantly slower than non-smokers (respectively, *p* < 0.031 and *p* < 0.042). The other ANOVAs did not report any significant effects.

A further one-way ANOVA was run using the attentional bias index (AB) as dependent variable and smoking status as fixed factor (current smokers, former smokers, and non-smokers). The difference between the smoking status groups was significant (*F*_(2,244)_ = 8.561, *p* < 0.000). In particular, Bonferroni *post hoc* analysis revealed that non-smokers were significantly faster than former-smokers (*p* < 0.002) and current smokers (*p* < 0.001).

Finally, a mixed-design ANOVA was run using stimulus (IC, CC, SC, NC) as within factor and smoking status (current smokers and former smokers) as between factor. As expected, we found a significant difference for the main effect stimulus (*F*_(1,244)_ = 93.053, *p* < 0.000), while the interaction stimulus × smoking status was not significant (*F*_(2,244)_ = 0.844, *p* = 0.470).

### Correlational Analysis

Current smokers’ response latency during the Emotional Smoke Stroop Task positively correlates with total impulsiveness, but this not true for former smokers and non-smokers. The association between response latency and impulsiveness in smokers is mainly explained by the motor impulsiveness (see Table 3): current smokers’ motor impulsiveness is positively associated with the response latency for incongruent words (*p* = 0.011), smoking-related words (*p* = 0.019), and neutral words (*p* = 0.025). Finally, no-planning impulsiveness correlates with NC (*p* = 0.029), while there is a negative correlation between NC and reward responsiveness in the BIS/BAS (*p* = 0.038). No statistical correlations were observed for former smokers and non-smokers (see Table 3).

**Table 3:** Correlations’ value (*r, p*) between BIS-11, BIS-BAS and response latencies in Emotional Smoke Stroop Task.

	Smokers				Former smokers				Non-smokers			
	CC	IC	SC	NC	CC	IC	SC	NC	CC	IC	SC	NC
**BIS-11**												
Total score	0.155	0.223*	0.255*	0.237*	0.065	0.049	0.022	0.044	−0.124	0.091	−0.042	−0.022
Non-planning impulsiveness	0.090	0.096	0.156	0.208*	0.032	−0.052	−0.021	−0.037	−0.048	0.153	0.023	0.048
Motor impulsiveness	0.139	0.287*	0.285*	0.236*	0.108	0.161	0.110	0.139	−0.262	−0.131	−0.182	−0.215
Attentional impulsiveness	0.110	0.202	0.202	0.157	−0.029	−0.015	−0.105	−0.077	−0.138	−0.020	−0.030	−0.028
**BIS-BAS**												
Drive	0.055	0.037	0.070	−0.035	0.017	−0.033	0.093	0.114	−0.018	0.017	0.049	0.045
Fun seeking	0.016	−0.061	−0.005	−0.082	−0.064	0.017	−0.009	0.006	−0.118	0.095	−0.023	−0.063
Reward responsiveness	−0.076	0.053	0.092	−0.201*	−0.107	−0.123	−0.086	−0.068	−0.030	0.050	0.043	0.035
BIS	0.034	0.180	0.127	0.121	0.044	−0.015	0.021	0.012	0.031	−0.109	−0.088	−0.028
BAS	−0.016	0.024	−0.010	−0.150	−0.083	−0.085	−0.034	−0.010	−0.080	0.142	0.046	0.024

*p < 0.05 (Bonferroni correction applied). CC, Congruent cues; IC, Incongruent cues; SC, Smoking cues; NC, Neutral cues.

Current smokers showed a positive correlation between total impulsiveness and number of false alarms (*p* = 0.025) and a negative correlation between total impulsiveness and response latency (*p* = 0.016). In addition, former smokers showed a positive correlation between the BAS and the number of false alarms (*p* < 0.001), and a negative correlation between total impulsiveness and response latency (*p* = 0.012). Other correlations were reported for singular subscales of BIS-11 and BIS-BAS in Table 4.

**Table 4:** Correlations coefficient between BIS-11, BIS/BAS and response latency and the number of false alarms in Go/no Go Task.

	Current smokers		Former smokers		Non-smokers	
Go/no go Task	False alarms	Latency	False alarms	Latency	False alarms	Latency
**BIS-11**						
Total score	0.208*	-0.376*	-0.087	-0.369*	-0.058	0.063
Non-planning impulsiveness	0.259**	-0.360*	-0.007	-0.277	0.103	0.067
Motor impulsiveness	0.201*	-0.214	-0.077	-0.376*	-0.128	-0.061
Attentional impulsiveness	0.049	-0.278	-0.165	-0.242	-0.067	0.122
**BIS-BAS**						
Drive	0.025	-0.227	-0.133	-0.229	0.243	-0.277
Fun seeking	-0.063	-0.236	-0.125	-0.141	-0.103	0.056
Reward responsiveness	-0.014	-0.349*	-0.202*	-0.253	0.281	0.001
BIS	0.052	-0.107	-0.104	-0.072	-0.284	0.041
BAS	-0.083	-0.318	-0.252*	-0.319	0.173	-0.085

*p < 0.05, **p < 0.01.

No statistical correlations were observed for non-smokers.

Finally, we performed a hierarchical regression model to test if the AB effect might be predicted by some psychological and/or behavioral variable. In details, we used the smoking status (current vs. former smokers) as dummy variable and years of smoking, number of daily cigarettes, in the first block, and then we added impulsivity in the second block and BIS/BAS subscale in the third. We found that the smoking-related AB was not predicted by any of the variables considered.

## Discussion

According to the Incentive Salience theory of addiction and PC theory, stimuli associated with tobacco cigarette smoking acquire high approach value (Robinson and Berridge, 1993). The increased salience of such stimuli results in an attentional bias, which may initiate cravings, urgency and substance use. The attentional bias has also been shown to predict relapse better than self-reports measures and other indexes (Cox et al., 2002; Waters et al., 2003a,b).

Coherently with this background, our findings confirm the presence of a bias toward smoking cues in current tobacco cigarette smokers. However, we also found a similar effect in former smokers and this evidence contrasts with some of the previous works (Bradley et al., 2003; Munafò et al., 2003; Waters et al., 2003a). Actually, there is still poor and contradictory evidence about AB in former smokers: the few studies present in literature used different methods to measure it (for example, Emotional Stroop task, Visual Probe task, and eye movements monitoring) and the samples size were generally small. Consequently, data are not always comparable, while some studies are not robust enough to support experimental hypotheses. Furthermore, Field et al. (2014) affirmed that while AB is a strong predictor of relapse during the short period after the interruption, its impact is unpredictable in the long term.

Our results suggest that former smokers’ attention might be modulated by smoking cues similarly to current smokers. Since the enrolled former smokers were abstinent on a long-term basis, the present study suggests that the AB persists and might influence cognitive processing also long after they stopped smoking. The presence of this bias might interfere with the ability to remain abstinent particularly in a stressful situation, as reported in other substances users (Field and Powell, 2007). Actually, different studies described the role of stress in tobacco smoking relapse as well, since negative affect, stress, and arguing with other people are often reported before they start smoking again (Marlatt and George, 1984; Baker et al., 2004; Shiffman and Waters, 2004). We argue that the AB might interact with negative emotions in promoting the desire and/or the urgency to smoke.

Furthermore, our study participants, independent of their smoking status (current, former or non-smokers) reported similar performance at the standard Stroop task and at the Go/no-Go task. Consequently, we may assume that the AB found in current and former smokers was not due to the impairment of general cognitive control functions, such as inhibition mechanisms. Previous studies on alcohol and cocaine abusers showed that there is an impairment effect of drugs on inhibition and that this effect is detectable at doses that do not lead to a global impairment in cognitive performance at a Go/no-Go tasks (Lane et al., 2007; Verdejo-García et al., 2007). Besides, physiological, motivation and attentional mechanisms seem to be interdependent, so that they all modulate the psychological value of a drug-cue (Kakoschke et al., 2017). In our study, the power of cigarettes to interfere with the cognitive processing seems to be associated only with smoking cues, which might be effective in increasing directly the power to grab the attention or by inducing inhibitory control failure. In particular, the performance at the Go/no-Go task did not suggest any general impairment of inhibition mechanisms in current and former smokers as remarked above. However, since deficient inhibitory control may also be considered a component of impulsivity (de Wit, 2009), we could expect some correlations between impulsivity level, cognitive mechanisms, and cigarette smoking. Actually, we found that current smokers present a significant correlation between impulsivity levels and performance at the Go/No-Go task. This evidence might suggest that smoking cues may reduce the ability of smokers to inhibit their responses in specific contexts. Although we did not find AB to be associated with impulsivity dimensions, our results about the relationship between impulsivity and inhibitory control in current smokers might suggest that the effect of the AB on craving, reported by previous studies (Grant et al., 1996; Hester et al., 2006; Ferguson and Shiffman, 2009), may be due both to the increased power of some stimuli to attract information and the inability of smokers to inhibit the responses. These results are coherent with other studies on cocaine, which reported that cocaine users with poor inhibitory control had also an intense bias toward cocaine-related words on the Emotional Stroop task (Liu et al., 2011). Since we found impulsiveness to be positively generally correlated to latencies at our Emotional Smoke Stroop Task, we might suggest that there is an association between AB and inhibition control deficit in tobacco smokers too. Consequently, our data suggest that impulsivity and AB are not directly associated, but that there might be a more complex relationship mediated by inhibitory control mechanisms.

More generally, we did not find any differences between current smokers, former smokers and non-smokers with respect to impulsiveness and BIS/BAS traits. Actually, previous studies failed to provide convergent data about this association (Doran et al., 2004). Some studies reported current smokers to be more impulsive, novelty seekers and less inhibited, suggesting that personality characteristics might be more important than other variables (Flory and Manuck, 2009). However, our results do not support this view. A possible explanation is that we collected data on a quite old sample made by smokers with a long history of smoking (having smoked for more than 10 years). Probably, persistence in smoking is not a function of impulsiveness, depending on other variables that sustain the smoking habit, often perceived as a safe and “natural” part of smokers’ lifestyle (Masiero et al., 2015). Persistence and dependence may be differently modulated by impulsiveness in younger smokers (Smith, 2017). This datum is further confirmed by the fact that neither the BIS/BAS scale was able to distinguish current from former smokers and non-smokers. Our participants have a similar approach and avoidant attitudes, and thus our data do not support the idea that smoking behavior is linked to a physiological predisposition to the search of gratification. In particular, impulsiveness and other induvial characteristics might be considered only as modulators of smoking behavior, since cognitive biases may activate the smokers’ wanting system independently of personality traits (Benowitz, 2010). Finally, we did not find any association between AB intensity and participants’ characteristics. In fact, AB was not associated with dependence level, number of daily cigarettes or years of abstinence.

From a clinical point of view, the present findings have important implications, suggesting that particular attention should be given to all cognitive mechanisms that sustain smoking, instead of focusing only on personality traits (Gorini et al., 2012; Gilardi et al., 2014; Masiero et al., 2016). This is particularly true for older smokers, who might want to stop smoking without being able to contrast the environmental solicitations. Furthermore, former smokers should be advised about the potentially detrimental effects of cognitive biases, providing them also behavioral strategies to counterbalance the correlated effects. Thus, it is important to take into account AB and more in general implicit cognitive measures (including working memory and executive function) in order to predict the success of treatment and/or to create tailored interventions (Wiers et al., 2002; Stacy and Wiers, 2010). In this vein, attention bias modification to avoid smoking-related stimuli might be a good strategy to help smokers quit, but further studies are needed to assess and to better define how to integrate it in clinical practice (Lopes et al., 2014).

A series of constraints limit the generalization of our results. First, our sample was not balanced as we had only a small group of non-smokers. Thus, the interpretation of our data, in particular, with regard to differences in impulsivity and BIS/BAS measures should be taken with caution. Second, we used as a measure of the AB a cognitive task, the Emotional Smoke Stroop task that cannot be considered particularly ecological and does not have strong internal reliability (Ataya et al., 2012; Field et al., 2014). In fact, Shiffman et al. (2015) affirmed that this as well other similar tasks are not particularly consistent, and that laboratory measures do not always correlate with the actual behavior in everyday life (Shiffman et al., 2015) so that more ecological methods are needed. For example, the ecological momentary assessment (e.g., diaries), which measure real-time data in the natural environment, might be considered a more accurate measure of affect, craving and other aspects of smoking, also able to predict smoking relapse during abstinence (McCarthy et al., 2006; Bujarski et al., 2015).

However, we argue that data coming from different methods and settings might help to further advance our knowledge of implicit cognition in tobacco cigarette smokers and the impact of this aspect on smoking’s trajectory (initiation, maintain and relapse). Consequently, we believe that research on tobacco smoking needs a wide-range approach.

The last concern is linked to the fact that having only behavioral data, we cannot support our finding from a neuro-functional point of view. Consequently, some of the issues raised are speculative. However, behavioral and neuroscientific data need to be integrated within a common and sound neuro-cognitive model, so to provide effective data both to researchers and health professionals. For these reasons, we believe that the research about AB should follow parallel pathways, without discarding data sources that proved to be useful, even though they are by their nature imperfect.

In conclusion, our results suggest and support future research, also integrating and advancing previous evidence that reported partial and contrasting data on small samples and on recent or even very recent former smokers.

## Ethics Statement

The Ethical Committee of the European Institute of Oncology approved the study. All enrolled participants were provided with full details about the study. All participants complied and signed the informed consent form. The study was in accordance with the principles stated in the Declaration of Helsinki (59th WMA General Assembly, Seoul, 2008).

## Author Contributions

GP, CL, MM, PM, and GV conceived and designed the study. GP coordinated the study. CL and GP acquired legal authorizations. MM managed the participants. Statistical analysis was performed by CL. Drafting and writing of the manuscript were handled by CL, MM, KM, and GP. All authors have read and approved the final manuscript.

## Conflict of Interest Statement

The authors declare that the research was conducted in the absence of any commercial or financial relationships that could be construed as a potential conflict of interest.
